# A Comprehensive Review of Biopolymer Fabrication in Additive Manufacturing Processing for 3D-Tissue-Engineering Scaffolds

**DOI:** 10.3390/polym14102119

**Published:** 2022-05-23

**Authors:** Nurulhuda Arifin, Izman Sudin, Nor Hasrul Akhmal Ngadiman, Mohamad Shaiful Ashrul Ishak

**Affiliations:** 1 Quality Engineering, Malaysian Institute of Industrial Technology, Universiti Kuala Lumpur (UniKL), Persiaran Sinaran Ilmu, Bandar Seri Alam 81750, Johor, Malaysia; nurulhuda@unikl.edu.my; 2 School of Mechanical Engineering, Faculty of Engineering, Universiti Teknologi Malaysia, 81310 UTM Skudai, Johor Bahru 81310, Johor, Malaysia; izman@utm.edu.my; 3 Faculty of Mechanical Engineering Technology, Universiti Malaysia Perlis, Kampus Pauh Putra, Arau 02600, Perlis, Malaysia; mshaiful@unimap.edu.my

**Keywords:** additive manufacturing, tissue engineering, biomaterials, scaffold, 3D printing

## Abstract

The selection of a scaffold-fabrication method becomes challenging due to the variety in manufacturing methods, biomaterials and technical requirements. The design and development of tissue engineering scaffolds depend upon the porosity, which provides interconnected pores, suitable mechanical strength, and the internal scaffold architecture. The technology of the additive manufacturing (AM) method via photo-polymerization 3D printing is reported to have the capability to fabricate high resolution and finely controlled dimensions of a scaffold. This technology is also easy to operate, low cost and enables fast printing, compared to traditional methods and other additive manufacturing techniques. This article aims to review the potential of the photo-polymerization 3D-printing technique in the fabrication of tissue engineering scaffolds. This review paper also highlights the comprehensive comparative study between photo-polymerization 3D printing with other scaffold fabrication techniques. Various parameter settings that influence mechanical properties, biocompatibility and porosity behavior are also discussed in detail.

## 1. Introduction

The failure of organs or tissues due to trauma or ageing is a primary concern in healthcare, as they are costly and devastating problems. Nowadays, technology transplantation from one individual into another has faced a significant challenge: to access enough tissue and organs for all patients. In addition, a problem exists with the immune system, which has a higher tendency to produce chronic rejection and destruction over time. These constraints have generated a need for a new solution to provide needed tissue. This has led to the development of tissue engineering (TE), which aims to create biological substitutes to repair or replace the failing organs and tissues [1].

Tissue engineering has gained more attention in the past decade, owing to its success in enabling tissue regeneration. The tissue-engineering field applies the knowledge of engineering, life, and clinical sciences toward solving the critical problems of tissue loss and organ failure. Tissue engineering also aims to produce patient-specific biological substitutes, to circumvent the limitations of existing clinical treatments for damaged tissue or organs. These limitations include the shortage of donor organs, chronic rejection, and cell morbidity [2].

**Figure 1:**
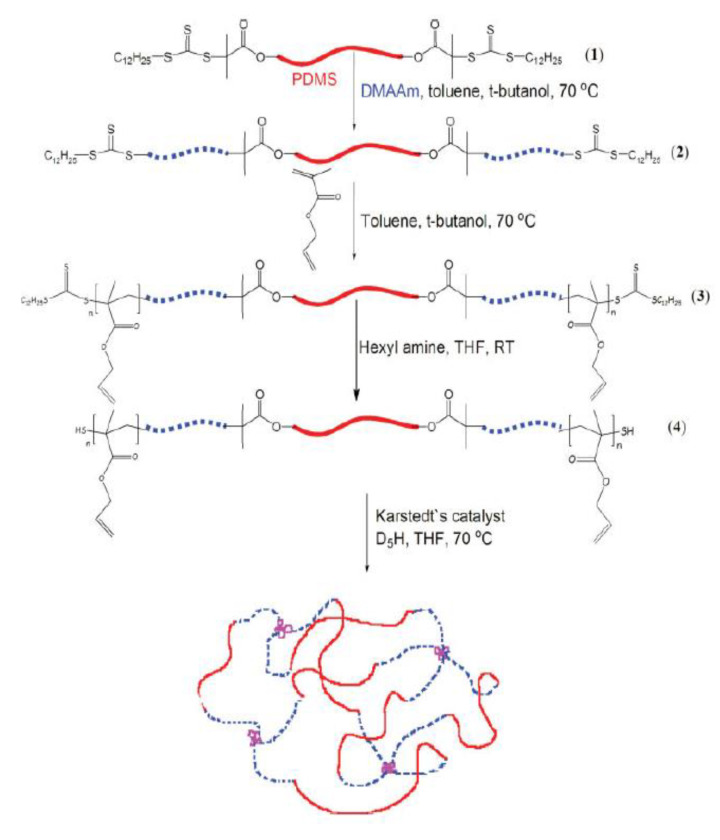
Strategy for the synthesis of APCN [4].

Tissue-engineering scaffold technology provides a temporary template from which to develop biological substitutes that restore, maintain, or improve tissue function or a whole damaged organ [3]. Tissue-engineering technology is unique in that it can establish three-dimensional environments for propagated cells and specific signaling molecules that can mimic native tissue environments. Typically, three groups of biomaterials—ceramics, synthetic polymers, and natural polymers—are used in the fabrication of tissue-engineering scaffolds. The scaffolds can be natural, synthetic or a hybrid of both. One example of a both natural and synthetic biomaterial is amphiphilic conetwork (APCN), which is useful for the controlled release of both hydrophilic and hydrophobic properties. Amphiphilic conetwork (APCN) gels are made up of hydrophilic and hydrophobic polymer chains that are covalently connected. The majority of APCN gels are made by the radical polymerization of telechelic macro-monomers having at least two polymerizable groups and a low molecular weight monomer [4,5,6]. End linking well-defined polymer chains with di-functional monomers produces APCN gels with a regulated structure as shown in Figure 1 [4].

On the other hands, biomaterials used in tissue engineering also can be categorized according to their origin by category: natural polymers (collagen, chitosan, hyaluronic acid, elastin and gelatin), synthetic polymers (poly(lactic acid) (PLA) and poly(glycolic acid) (PGA), polycaprolactone (PCL)), ceramics (HA, TCP and biphasic calcium phosphates) and metals (magnesium and nickel alloy) [7,8,9,10].

Tissue-engineering techniques have the potential to create tissues and organs. They involve the in-vitro seeding and attachment of human cells onto a scaffold. The cells then proliferate, migrate and differentiate into the specific cell type to repair tissue [11,12]. Therefore, the choice of scaffold is crucial to enable the cells to behave in the required manner, to produce tissues and organs of the desired shape and size. A useful tissue-engineering scaffold should fulfil the biological and mechanical requirements of the target tissue. The scaffold should: have a suitable microstructure to promote cell proliferation, be contained within an open-pore geometry with a highly porous surface that enables cell ingrowth, have a proper surface morphology and be made from biomaterials with a predictable rate of degradation with a nontoxic degraded material [2].

During the development of the scaffold, the primary aim is to imitate the structural and mechanical properties of bone as close as possible. Thus, the scaffold fabrication technique should be flexible, to build scaffold architectures with biomimetic designs. Generally, conventional methods are used to construct tissue-engineering scaffolds. There are various traditional methods used to construct tissue-engineering scaffolds, including the molding technique, solvent casting and particulate leaching, gas foaming, and electrospinning. Although a lot of conventional fabrication methods can be used to produce scaffolds, unfortunately, each of these methods has their own limitations as they are not able to precisely control the internal topology and architecture [13,14]. To the best of our knowledge, none of the traditional methods is satisfactory to produce scaffolds with a control dimension architecture, porosity and faced the difficulty for mimicking the biological function of natural tissue [13,14,15,16].

As an alternative to conventional scaffold-fabrication methods, additive manufacturing techniques have recently been developed in tissue engineering, such as a rapid prototype by which a 3D scaffold is fabricated by laying down multiple, precisely formed layers in series [17]. Subia et al. (2010) claimed that the rapid-prototype technique (RP) has drawn tremendous attention with its potential to overcome most of the limitations faced by conventional techniques for the fabrication of 3D scaffolds [18].

This review provides an overview of the advantages and limitations offered by the additive manufacturing process (AM), specifically in the photo-polymerization 3D printing technique compared to other conventional methods. The overview includes their advantages and limitations regarding mechanical properties and the internal architecture porosity of fabricated scaffolds. The potential of the photo-polymerization 3D printing technique in the fabrication of tissue-engineering scaffold hydrogels is also discussed detail.

## 2. Concept of TE Scaffold

Over two decades, many works have been carried out to develop potentially applicable scaffolds for tissue engineering. The scaffolds are designed in three dimensions (3D), with a porous solid structure to perform some or all of the following functions: (i) promote cell–biomaterial interactions, cell adhesion, and ECM deposition; (ii) permit sufficient transport of gases, nutrients, and regulatory factors to allow cell survival, proliferation, and differentiation; (iii) biodegrade at a controllable rate that approximates the rate of tissue regeneration under the culture conditions of interest; (iv) provoke a minimal degree of inflammation or toxicity in vivo; and (vi) contain the porous interconnected structure that is necessary to allow the spread of waste products from the scaffolding [19,20].

Scaffolds can be tough, as a mimic of a physiologic environment that serves to promote proper cell proliferation, differentiation and organization; all cell migration and interaction is often greatly influenced by the local environment [21]. In tissue engineering, researchers have designated the substitution of a native ECM as a “scaffold”, “template”, or “artificial matrix”. The scaffold provides a three-dimensional (3D) ECM analogue that functions as a template required for the infiltration and proliferation of cells into the targeted functional tissue or organ [22,23]. The concept of a tissue engineering scaffold involves the in-vitro seeding and attachment of human cells onto a scaffold. These cells then proliferate, migrate and differentiate into the specific tissue [11] and recover damaged tissues [12].

One of the more promising approaches in tissue engineering is to grow cells on a biodegradable scaffold that mimics the function of the natural extracellular matrix, providing a temporary template for the growth of target tissues [15]. The extracellular matrix (ECM) is the optimal support for tissue engineering, as it provides the perfect chemical composition, surface topology and physical properties experienced by cells in vivo [24]. The use of ECM derived from decellularized tissue is increasingly frequent in regenerative medicine and tissue-engineering strategies, with new applications including the use of three-dimensional ECM scaffolds [24]. One of the principal methods behind tissue engineering involves growing the relevant cells in vitro into the required three-dimensional (3D) organ or tissue. However, cells are difficult to grow in the favored 3D orientations and formed anatomical shape of the tissue. Instead, they randomly migrate to form a two-dimensional (2D) layer of cells. However, 3D tissues are required, and this is achieved by seeding the cells onto porous matrices to enhance the cells’ attachment and colonization [13,14,25].

### Requirement of TE Scaffold

Several requirements are identified as crucial for the production of tissue engineering scaffolds. Most of the researchers have summarized an ideal scaffold as having the following characteristics: (i) a scaffold should possess interconnecting pores of an appropriate scale to favor tissue integration and vascularization [26,27], (ii) a scaffold should be made from material with controlled biodegradability so that tissue will eventually replace the scaffold [15,26,28], (iii) have appropriate surface chemistry to favor cellular attachment, differentiation and proliferation [29], (iv) possess adequate mechanical properties to match the intended site of implantation and handling [28,30], (v) should not induce any adverse response [28], (vi) be easily fabricated into a variety of shapes and sizes [15,28], and (vii) must facilitate the ingrowth of tissue and possibly allow for the inclusion of seeded cells, proteins and/or genes to accelerate tissue regeneration [26,28]. All of these highlighted properties of a tissue engineering scaffold are important, to ensure the ability of the scaffold to be metabolized by the body, allowing it to be gradually replaced by new cells to form functional tissues.

In addition, the criteria for choosing materials as biomaterials in biomedical applications are based on their material chemistry, molecular weight, solubility, shape and composition, hydrophilicity/hydrophobicity, degradation of water absorption, and erosion mechanism [31]. The scaffold should have the mechanical strength needed for implantation and an appropriate strength that can influence the biostability of implants. The porosity and pore size of a supporting 3D scaffold is vital for tissue regeneration [27,32]. A large surface area also favors cell attachment and growth. Other than that, highly porous scaffolds are desirable for the diffusion of nutrients and waste products from the implant [33]. Hydrophilicity is also an essential factor need to consider. It is because hydrophilicity will enhance cell growth and proliferation of 3D scaffolds, as discussed previously [15]. Therefore, due to the important character and behavior of mechanical and porosity scaffolds, a nanofiber material is most suitable for nano-based scaffolding systems with the appropriate mechanical integrity, pore size, and hydrophilic property that will provide an excellent potential for tissue engineering scaffolds.

## 3. Fabrication of 3D TE Scaffolds

Currently, there are two broad categories of scaffold fabrication methods which are the conventional and advanced processing methods (additive manufacturing). The fabrication of tissue engineering scaffolds commonly involves traditional techniques such as (i) solvent casting, (ii) particulate leaching, (iii) electrospinning, (iv) phase separation, (v) extrusion deposition, (vi) pressing, (vii) freeze drying, and (viii) gas foaming [34,35,36,37]. Even though these methods have been extensively studied and optimized, they still have a lot of limitations.

There are critiques concerning the practicality of conventional methods. These methods were identified as techniques incapable of precisely controlling pore size, pore geometry, pore interconnectivity, and the spatial distribution of pores to allow construction of internal channels within the scaffolds, as argued by Zhu and Che (2013) [17]. In addition, several of these techniques are contingent upon using organic solvents with inherent biocompatibility when using a toxic solvent that may be toxic to the cells if they are not wholly and adequately removed [1,34].

The revolutionary technology of rapid prototyping in the additive manufacturing process offers potential and opportunities for manufacturing to fabricate 3D materials with optimized properties and multi-functionality. RP is also called the solid free-form technique or additive manufacturing (AM). This technique is a more advanced technique for scaffold fabrication. It is a computer-controlled fabrication technique that can rapidly produce a 3D object by using the layer manufacturing method. The RP technique generally comprises the design of a scaffold model by using computer-aided design (CAD) software [18,36].

There are numerous benefits offered by this rapid-prototype technology, such as ease of use, reliability, cost-effectiveness, and the diversity of the compatible materials [33]. Rapid-prototype techniques also hold much promise over conventional methods in terms of part consistency, design repeatability and the control of scaffold architecture such as pore size, porosity, surface area and the external shape of the scaffold architecture. The control over scaffold architecture is particularly important, as a TE scaffold mimics the original environment organ to regenerate damaged tissues [1] successfully.

The comparative study between fabrications techniques of 3D tissue engineering scaffolds is discussed detail in Table 1. From the summarized reviews on various fabrication techniques of 3D tissue engineering scaffolds, the rapid-prototyping method has lots of advantages to fabricate an excellent profile of biocompatibility properties with a more accurate scaffold architecture. This technique also promises the capability of mimicking the extracellular matrix (ECM) in the human body. Even though pure-polymer products built by rapid prototyping lack in strength, the technique of rapid-prototype polymer composites has solved these problems by combining the matrix and reinforcements to achieve high mechanical performance and excellent functionality.

### 3.1. Additive Manufacturing in TE Scaffold Fabrication

Additive manufacturing, also known as rapid prototyping or 3D-printing technologies in tissue engineering, has been growing in recent years. As pointed out by Bose et al. (2013), among the different technology options for tissue engineering scaffolds, the rapid-prototyping technique is becoming popular due to the ability to print porous scaffolds with a designed shape, controlled chemistry and interconnected porosity [62]. Wang et al. (2017) claimed that rapid prototyping can fabricate complex composite structures of tissue engineering scaffolds without the typical waste, compared to traditional methods. The size and geometry of composites can also be precisely controlled with the help of computer-aided design in the rapid-prototyping process [62]. Thus, tissue engineering scaffolds fabricated by rapid prototyping will have a higher performance.

Rapid prototyping is an additive manufacturing technique that builds the objects piece by piece, using only the material that will become part of the object and avoiding loss of material in the process. The technology of the 3D printer is tied to that of computer-aided design—CADs software—where the design of the object is made [55]. It is based on 2D cross-sectional data obtained from slicing a computer-aided design (CAD) model of the object [37]. Rapid-prototyping technologies can be divided into five main groups according to the working principles used to produce 3D objects. The main rapid-prototyping technologies developed within few years are (i) the photo-polymerization technique, (ii) fused deposition modeling (FDM), (iii) selective laser sintering (SLS), (iv) 3D printing (3DP), and (v) bioprinting (3D plotting or direct writing) [63].

Table 2 summarizes a detailed review of advantages and disadvantages for each rapid-prototyping technique that is useful for tissue engineering scaffold fabrication. Although there are a lot of different advanced technology approaches to fabricating scaffolds’ 3D structures, each of them has its limitations. Some of the techniques have a limitation when trying to mimic the biological function of natural tissue, due to the difficulty of finely controlling the scaffold architecture, dimensions, and porosity [64]. Chia and Wu (2015) claimed that the selection of a fabrication technique depends upon the materials of interest, machine limitations, and the specific requirements of the final scaffold. Other than that, design architecture is important for the structural, nutrient transport and cell–matrix interaction conditions of tissue engineering scaffolds [58].

#### 3.1.1. Photo-polymerization 3D Printing

There is a critique concerning the importance of considering the porosity and architecture of an engineering scaffold. As pointed out by Annabi et al. (2010), the porosity of a scaffold plays an important role in directing tissue formation and function. The authors suggested a substantial amount of scaffold porosity is often necessary to allow for homogeneous cell distribution and interconnection throughout engineered tissues. In addition, increased porosity can have a beneficial effect on the diffusion of nutrients and oxygen [77]. Based on a review study, Mondschein et al. (2017) suggested that photo-polymerization via the stereolithography (SLA) technique will allow a greater control of the tissue scaffold’s dimensions and features, compared to other additive-manufacturing techniques. The SLA technique can also precisely control the architecture and features of the scaffold, which offers a great benefit to regenerative medicine, whether to construct repeatable identical scaffolds or to fabricate patient-specific templates [76].

In the SLA approach, resolution is inversely related to print speed. Parts with a feature size down to 10 µm can be formed with z-axis print speeds of 25–1000 mm/min, which take several hours with conventional SLA techniques [78]. To overcome the limitation of SLA printing speed efficiency, another trend of the photo cross-linking 3D printing field is the emerging use of digital-light-projection (DLP) technology [79]. Surface patterned exposure from digital-light-projection (DLP) sources, and the high-power LED sources used, allows any selected portion of the entire x/y workspace to be exposed simultaneously to dynamic writing with a condensed laser beam. Even though high laser scanning velocities are employed in the SLA approach, the ability to simultaneously photo cure all portions of a given slice with the DLP technique significantly speeds up the cycle times between layers [78,79,80]. Thus, it allows control of spot-to-spot (lateral) and interlayer (vertical) binding and improves the resolution of printed parts [81].

#### 3.1.2. Stereolithography (SLA)-Based 3D Printing

Stereolithography-based 3D printing was developed by 3D systems in 1986 and is the first commercially available solid freeform (SFF) technique [82]. SLA is a particularly versatile manufacturing technique for the freedom of designing structures. In the biomedical field, the development of SLA technology has led to the fabrication of mold-assisted implant fabrication, aids for complicated surgery, and fabrication of tissue engineering scaffolds [83,84]. The manufacturing of 3D objects by SLA is based on the spatially controlled solidification of a liquid resin by photo-polymerization [85]. SLA allows the fabrication of parts from a computer-aided design (CAD) file. The CAD file describes the geometry and size of the parts to be built. This designed structure is (virtually) sliced into layers of the thickness that is to be used in the layer-by-layer fabrication process [82,86].

The SLA technique based on free-radical photo-polymerization is an efficient method for converting a liquid prepolymer resin into a solid polymer network under light exposure [87]. In free-radical chain-growth photo-polymerization, a photoinitiator absorbs light either in the UV or visible light range, which excites the photoinitiator molecules into a high-energy radical state. The initiator radicals interact with precursor molecules, forming the primary radicals that initiate the polymerization reaction. Chain-growth polymerization then propagates until a complex crosslinked network is formed in a process called photo crosslinking [88].

The SLA process can be divided into two major categories, which are the projection and laser-scanning types. The scanning-type stereolithography process uses a UV laser beam to scan and cure the surface of the resin layer by layer. On the other hand, in the projection-type stereolithography process, a digital light projection (DLP) is utilized to project a whole cross-sectional area of mask projection on the resin surface [81]. The scanning-type stereolithography apparatus consists of a bath to be filled with a liquid photocurable resin, a laser source (commonly, UV light), a system that controls the XY-movement of the light beam, and a fabrication platform that permits movement in the vertical plane [79]. In the bath configuration, the UV beam traces a 2D cross-section onto a base submerged in a tank of liquid photoactive resin that polymerizes upon illumination. After completion of the 2D cross-section, the UV beam begins the addition of the next layer, which is polymerized on top of the previous layer. In between layers, a blade loaded with resin levels the surface of the resin to ensure a uniform layer of liquid prior to another round of UV light exposure. This process is repeated, slice by slice, until the 3D object is completed [89]. After the structure is completed, the un-polymerized liquid resin is removed by draining and post-curing converts any unreacted groups.

#### 3.1.3. SLA versus DLP 3D Printing for TE Scaffold Fabrication 

Digital light processing (DLP) is identical to SLA except for the light source: a projector is used to cure an entire layer at a time (Table 3). Instead of using a UV laser, a DLP projector is used to project the entire cross-sectional layer of the 3D structure [90]. The photosensitive resin is exposed to the light through patterns on a digital mirror device. The exposed parts are cured, and one layer is finished. Then, the platform raises a layer, and the next exposure starts [91], projecting the image of a layer of the part to be built onto the photosensitive resin allows one to fabricate one layer at once, a fact responsible for the high building speed at a significantly lower cost of equipment achieved with these machines [92,93]. For the fabrication of 3D parts, the CAD model was sliced, and every slice was projected onto the bottom layer of the resin tank by a micro-mirror array. Here, the first layer of the light-sensitive resin cured in a few seconds. The polymer adhered to the z-stage, which was then moved upwards [94].

There are two types of projection-type stereolithography DLP process, namely, free surface and constrained surface. In free surface (top-down stereolithography), the layer is cured on the photopolymer from the surface towards the bottom of the vat. On the other hand, in constrained-surface (bottom-up) stereolithography, the layer is cured through the bottom of the vat, so that the printed structure does not adhere to the substrate. It causes the curing of liquid resin to be sealed from the oxygen-rich environment. By eliminating the oxygen inhibition effect, the liquid photopolymer resin can be cured faster, which offers an advantage over the free surface-based system [95].

According to a study conducted by Low et al. (2017), Groth et al. (2014), and A. Woesz (2008), the DLP system has the same general advantages and disadvantages as the SLA method [90,92,96]. In principle, the main advantages of DLP over SLA in the context of scaffold fabrication are that DLP 3D printing does not use a laser, which reduces the system costs significantly. It also has a higher build speed due to the exposure of one layer at one time. A detailed study of the specifications for SLA and DLP 3D printing are summarized in Table 3.

**Table 3:** Comparison between laser-scanning-type SLA and projection-type SLA (DLP) 3D printing.

Technique	Resolution µm	Light Source	Advantages	Disadvantages	Ref.
Laser-scanning-type SLA	200–300	UV	NA	Slow	[90,92,96,97]
Projection-type SLA (DLP)	15–100	Projector	Higher speed than SLA Low cost	Lower light intensity	

Compared with other advanced manufacturing techniques, SLA shows its superiority in its high resolution. The higher the resolution at which a part can be built, the smaller will be its maximum size. In order to achieve a high resolution, SLA requires a high level of control over the layer thickness being crosslinked. In the SLA technique, control of the thickness of the layer is crucial. The study of curing depths in photo-polymerization is an important aspect of the curing process because it affects the final dimensions of the cured sample. Therefore, it is important to optimize the cure for these systems.

The smallest feature size that can be produced depends on the resin and setting parameters. Melchiorri et al. (2016) examined techniques and materials developed for the DLP printing of vascular tissue engineering scaffolds utilizing poly (propylene fumarate) (PPF). The researcher found that the mechanical properties of the 3D-printed structure relied largely on the amount of post-printing time to radiation, which increased polymer cross-linking [98]. In line with this, Valentincic et al., (2017) proved that the setting parameter of DLP is important to produce the optimum result. The researcher argued that there are three main printing parameters in DLP printing that needs to take consideration which is curing time, layer thickness and time between the consecutive exposures. It was recorded in their study that an increased exposure time significantly increased illumination intensity on the whole of the projection surface [99].

Although the principles of projection or laser-scanning processes are similar, the effects of process parameters on curing the polymeric-photo material can be quite different. The light in the projection-type process and the UV-scanning type can be different in energy densities due to various control parameters such as curing time and scanning speed, which will correspond to the varying degrees of polymerization. Therefore, it is essential to determine the critical energy density by the UV projector or laser, to form a solid network.

#### 3.1.4. Influence Process Parameter

A previous study by Chong et al. (2016) and Tureyen et al. (2015) agreed that the most challenging in photo-polymerization 3D printing is to control curing issues. The researchers reported that light intensity has a significant role in the curing depth of the resin. The curing depth and width of printed parts can be controlled by adjusting the curing time or laser-scanning speed, respectively: the energy density is increased by extending the curing time and lowering laser-scanning speeds. At the same energy density, it is shown that the projection-type SLA process obtains a larger curing depth than the laser-scanning type due to the difference in intensity between both the systems [81,100]. Table 4 summarizes a list of recent references on the research work performed to produce TE scaffolds by using photo-polymerization processes. In view of Table 4, the influences of the resin used and parameter setting are also studied.

Based on the summarized review of previous research in Table 4, it is shown that different input setting parameters and the type of resin used will give a different response on the cured thickness of solidified resin, mechanical properties, biocompatibility, and the porosity of the scaffold. Wang et al. (1996) found that the curing degree is approximately proportional to the intensity of the light source, scanning speed and type of resin used. The final degree of the cure of a photo-polymerization-based 3D-printing prototype is determined by the combination of all of these factors. Researchers declared that if the intensity is increased, the curing degree will be increased. This is because, by using a high intensity power source, the resins will have more cross-linking. When the scan is fast, the exposure energy in a unit area is less; thus, the curing degree will be low [105].

In the same context, some researchers have studied the curing process of photo-polymerization resin for SLA 3D printing. Most of the research revealed that the curing phase in SLA is important for further solidification and, thus, causes an enhancement in the prototypes’ mechanical properties. Thermal and the heating effects during the curing process also led to the existence of shrinkage and distortion within the structure of a cured resin [105,111]. As compared to the laser-scanning SLA process, projection-based SLA (DLP) has a lower light intensity and resulted in less polymerization, causing the printed parts to be deformed due to inhomogeneous curing [97]. However, to the best of our knowledge, there are only a limited numbers of studies performed on the investigation effect of curing time via DLP.

## 4. Materials for Photo-Polymerization 3D Printing TE Scaffold

The selection of biomaterials plays a crucial role in tissue engineering. The materials should obtain interactions with cells to enhance cellular attachment, proliferation, and new-tissue formation. However, the limitation of biocompatible resins with suitable SLA processing has often been considered as the main disadvantage of this method. Resins utilized in this process should be a liquid that solidifies quickly on illumination with light. The resin used must not only exhibit fast photo-crosslinking kinetics but also enhance adhesion and cell proliferation and has proper mechanical properties after crosslinking [68]. Other than that, the SLA process requires resin that has a melting temperature™ below room temperature and a glass transition temperature (Tg) low enough to maintain the polymer in a liquid-like state, to allow chain mobility. The low viscosity of resin used is also important to obtain optimum cure rates, thus decreasing overall construction times [64]. 

There are differences between polymers and resins as shown in Table 5. It can be summarized that polymers have large molecules with repeating structural units of monomers, while resins are an organic material that naturally forms in plants. Resins have low molecular weights, whereas polymers have large molecular weights. Additionally, resin is a viscous liquid that can be clear or dark brown in color, whereas polymers can be solid or liquid. 

**Figure 2:**
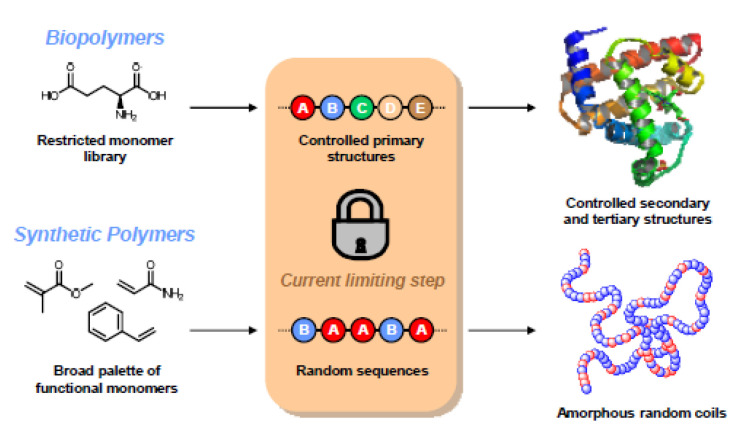
A schematic representation of controlled comonomer sequences: a key-step toward highly organized polymer-based materials [88].

In the photo-polymerization process, the process is driven by a chemical reaction that produces free radicals when exposed to specific wavelengths of light. The problem with this photo-polymerization process is that the created free radicals can cause damage to the cell membrane, protein and nucleic acids. Therefore, it is important to find a cytocompatibility photo-initiator resin for the SLA 3D printing method [68]. One of the remaining big basic issues in polymer science is controlling comonomer sequences in synthetic polymerization techniques. Modern synthetic processes, in fact, do not allow for accurate control of polymer microstructures. This is particularly true for radical chain-growth polymerizations, which frequently result in statistical comonomer inclusion in polymer chains as illustrated in Figure 2 [88].

The first resins developed for use in SLA are based on low-molecular-weight polyacrylate and epoxide or viny ester-based resin [112], which form glassy networks upon photo-initiated polymerization and cross-linking [82]. When preparing biomedical implants, the use of epoxy- or acrylate-based resins is limited. The advantages of these materials include several useful properties, such as low viscosity, high photosensitivity, controllable mechanical properties, and relative insensitivity to temperature and humidity changes. However, its disadvantages are still noticeable. These materials are usually not biocompatible or biodegradable [113] and have poor dimensional stability and high-volume shrinkage during the post-curing process [114]. 

In biomedical applications, the photosensitive resin used should integrate without having a toxic effect on the living system, and be biodegradable and bioactive for biomedical applications [112]. Current research is tremendously focused on the development of light-curable and highly biocompatible resin for SLA, such as poly(ε-caprolactone) (PCL) based materials, poly(propylene fumarate) (PPF), poly(D,L-lactide) (PDLLA) resins, and polyethylene glycol (PEG)-based resins, polyglycolic acid (PGA), and polylactic-co-glycolic acid (PLGA) [115,116,117,118,119] which are resins that are usually composed with a photoinitiator, polymerizable oligomers, and a reactive or non-reactive diluent and additives [120]. 

The amorphousness of poly(D, L-lac-tide) (PDLLA) has successfully been applied in resorbable bone-fixation devices clinically [121]. A PDLLA-based resin was developed using ethyl lactate with a non-reactive diluent such as methyl methacrylate, butane-dimethacrylate and N-vinyl-2-pyrroli (NVP) [119]. PDLLA resin material has a glass transition temperature of approximately 55 °C and an elasticity modulus up to 3 GPa, making it one of the biodegradable polymers with mechanical properties that closely similar to the E-modulus of bone (3 to 30 GPa) [122]. PDLLA-based materials by SLA would optimize structures for bone-tissue engineering with regard to mechanical properties and cell seeding [123]. Other than that, Poly(D,L-lactide) (PDLLA) also has been successfully applied in resorbable bone-fixation devices clinically [124] and is well-suited for bone tissue engineering [125]. However, polylactides have occasionally been found to undergo rapid bulk degradation. Degradation products of these materials reduce local pH, accelerating the polyester degradation rate and leading to a localized acidosis and inflammation [126,127].

In 2007, Lee et al. successfully synthesized and modified poly(propylene fumarate) (PPF) by adding diethyl fumarate (PPF/DEF) resin using the SLA process. PPF is a biodegradable and UV-curable material, as an SLA resin. Since synthetic PPF has a high viscosity, it cannot be used in SLA systems directly. Therefore, DEF was added to reduce the viscosity, and photoinitiator dimethoxy phenyl acetophenon (DMPA) [102] or bisacrylphos-phrine oxide (BAPO) [118] was used to initiate the UV polymerization of PPF/DEF. The researcher proved the mechanical properties and cell adhesion of the PPF/DEF scaffold has good potential as a bone scaffold for tissue engineering and the finding in this research showed that the measured mechanical properties of the PPF/DEF scaffold were similar to those of human trabecular bone, which proved that the possibility of the PPF/DEF scaffold as a bone scaffold [102]. 

Another one of the suitable resins is a photocrosslinkable poly (e-caprolactone) (PCL), which has been studied by Eloma et al. (2011). Poly (e-caprolactone) (PCL) is a highly biocompatible elastic polymer with a low melting temperature. The researcher developed a PCL resin and applied it using SLA, without any additional solvents required during the structure-preparation process. Results recorded the photo-crosslinkable and highly interconnected pore network, and biodegradable PCL resin for the solvent-free fabrication of tissue engineering scaffolds by stereolithography with no observable material shrinkage in 3-D scaffolds produced [118]. 

Even though there are many types of synthetic polymeric biomaterial which are noteworthy in use, most researchers declared that poly (ethylene) glycol (PEG) resin was widely used in biomedicine because of its excellent biocompatibility and hydrophilicity efficiency, making it appropriate for biomedical applications [110,128,129,130,131,132,133,134,135]. Due to its features as non-toxic, non-immunogenic and being readily removed from the body, PEG synthetic hydrogel polymer is widely used in tissue regeneration. The hydrogel-based scaffolds provide an environment with a high water content, enabling high cell-encapsulation densities [13,79,93]. These hydrogels are permeable to oxygen, nutrients and other water-soluble metabolites and have a smooth consistency that makes them soft-tissue-like [127]. Further, because PEG hydrogels are water-soluble, their chains can be easily modified by photoreactive and cross linkable groups such as acrylates or methacrylates with the high crosslinked hydrophilic polymer network that is recommended for use in a diversity of biomedical applications. These hydrogels exhibit 1–100 kPa of mechanical strength [112] and offer flexible, tunable mechanical properties and are soft due to being intermolecular crosslinked to form a stability similar to tissue of the body. 

The list of photocurable resin material SLA that have been studied in the development of scaffolds is compiled in Table 6. Based on the review, it can be expected that the different selection of resin materials used in a photo-polymerization 3D printing TE scaffold greatly influences the mechanical properties of the scaffold produced.

### Commercial Available Materials and Global Market in the Future Prospective

Biomaterials used in tissue engineering can generally be categorized according to their origin by category: natural polymers, synthetic polymers, ceramics and metals. Each of these biomaterials has particular benefits and disadvantages. The metals group is not a suitable option for applications in tissue engineering scaffolds as they are not biodegradable, and their processability is very limited. Ceramic scaffolds have high mechanical stiffness (Young’s modulus), low elasticity and a brittle surface. They are highly biocompatible due to their chemical and structural likeness to the bone-mineral phase. However, their clinical applications for tissue engineering are restricted due to their fragility, implantation difficulty [64]. For these reasons, polymeric biomaterials have become increasingly common, due to their biodegradable and biocompatible properties. The group of polymer biomaterials is very efficient because they can be produced with a tailor-made design and their degradation characteristics can be controlled by varying the polymer itself or the individual polymer structure [103].

Polymeric scaffolds play very significant roles in tissue engineering as they are intended to bring cells together and regulate their function to enhance tissue growth [41]. Due to their distinctive characteristics such as a high surface-to-volume ratio, good in biodegradability and mechanical properties, polymeric scaffolds draw tremendous attention. They also offer various advantages of bio-compatibility, chemistry versatility and biological characteristics that are important for tissue engineering and organ substitution [31]. 

Biodegradable scaffolds can actually be fabricated as naturally derived and synthetic. Previous studies indicate that most natural biomaterials, such as collagen, chitosan, hyaluronic acid, elastin and gelatin, are suitable for the development of liver, nerve, bone and heart tissue [6]. Natural polymers can be classified as the first biomaterials to be used clinically in tissue engineering scaffolds. They can be categorized as enzymes (cellulose, amylose) or polynucleotides (DNA, RNA) [31]. Natural polymers of biomaterials have excellent biocompatibility and potential viability. They also establish bioactive characteristics and better cell interactions, which enable them to improve the efficiency of the cells in the biological system. However, one of limitations of natural polymers is their poor mechanical properties, as the natural materials have limited physical and mechanical stability. Therefore, they are not preferred for load-bearing scaffold applications. By using synthetic polymers, the issues associated with natural polymers can be eliminated by using synthetic polymers, as their physical and chemical properties can be changed and repeatedly produced [6].

Synthetic polymers are incredibly beneficial in the biomedical industry due to their properties such as chemical modification, excellent biocompatibility, high versatility, optimal mechanical properties, porosity, degradation time and mechanical characteristics that can be tailored for targeted applications under control [138,139]. Other than that, synthetic polymers cost less than natural polymers for biological scaffolds, which can be manufactured in huge quantities and have a significant shelf life. Synthetic polymers are commonly split into two groups: non-biodegradable and biodegradable. In tissue engineering, the most frequently used biodegradable synthetic polymers for 3D scaffolds are poly(lactic acid) (PLA) and poly(glycolic acid) (PGA), polycaprolactone (PCL), poly(lactic-co-lycolide) (PLGA) copolymers and so on; while an example group of non-biodegradable polymers includes polyvinyl alcohol (PVA), polyhydroxyethyl methacrylate (PHEMA), poly(N-isopropylacrylamide) (PNIPAM) and others. 

## 5. Biocompatibility Test

Biocompatibility is a term that covers many aspects of a material, including its physical, mechanical, and chemical properties, as well as its potential in cytotoxicity, therefore there are no significant injuries or toxic effects on the biological function of cells which can possibly inhibit the beneficial properties of a cell ECM scaffold. The term biocompatibility is also defined not only by the lack of the cytotoxicity of a biomaterial but also by the bio-functionality of the material, which enables it to support cell–biomaterial interactions [133,140]. In tissue engineering applications, a scaffold must be non-toxic and biologically compatible so that cells can safely adhere, proliferate, and differentiate within the scaffold [134]. Toxicity and biocompatibility tests are needed to evaluate a scaffold material in facilitating cell proliferation and differentiation, secreting an extracellular matrix and carrying biomolecular signals for cell communication [135]. In measuring biocompatibility, there are several varieties of tests that are currently used to identify whether new materials are biologically acceptable. These tests are classified as the in-vitro, in-vivo, and usage tests.

The most common biocompatibility test for a tissue engineering scaffold is an in-vitro test. The testing is performed outside a living organism, requiring the placement of a material or a component of a material in contact with a cell, enzyme, or some other isolated biological system. In-vitro tests can be roughly subdivided into those that measure cytotoxicity or cell growth. The cytotoxicity tests assess cell death caused by a material by measuring cell number or growth before and after exposure to materials. Membrane-permeability tests are used to measure cytotoxicity by the ease with which a dye can pass through a cell membrane, because membrane permeability is equivalent to or very nearly equivalent to cell death [136]. Some in-vitro tests for biocompatibility use the biosynthetic or enzymatic activity of cells to assess cytotoxic response. A standard method to analyse the biocompatibility properties describes details in the standard testing ISO 10993, which includes a series of guidelines for analyzing biocompatibility and medical devices.

On the other hand, in the field of biocompatibility, some scientists questioned the usefulness of in-vitro and animal tests due to the apparent lack of correlation with usage tests and the clinical history of materials. Furthermore, barriers between the material and tissues may exist in usage tests or clinical use but may not exist in the in-vitro or animal tests. Thus, it is important to remember that each type of test has been designed to measure different aspects of a biological response to a material, and correlation is not always to be expected. Among the biocompatibility tests, the in-vitro tests have several significant advantages over other types of biocompatibility tests (Table 7). In-vitro tests are relatively quick with a lower cost than animal or usage tests, are well-suited to large-scale screening, and can be tightly controlled [136].

The concept of correlation between the in-vitro and in-vivo tests has been reported, confirming the advantage of in-vitro tests as a system to select the biomaterials. In cytotoxicity testing, the same type of cells is used. The testing of scaffolds must have cells derived from the tissue origin to ensure a better simulation of the clinical situation [137]. For example, scaffolds derived from orthopedic tissues should be tested on osteoblasts or osteoblast-like cells; cardiovascular-derived scaffolds should be assayed using endothelial cells or cardiomyocytes. For in-vitro cytotoxicity screening, the recommended testing methods include (i) indirect contact assay or the extraction method, and (ii) a direct contact assay. The guidelines on inspecting the biocompatibility of materials for medical applications is set in the International Standard ISO10993 (International Organization for Standardization, 1999), with priority being given to cell-culture-based in-vitro tests using both the direct and indirect contact approaches.

### 5.1. Indirect Contact Assay

The indirect contact assay technique applies cell counting, dye-binding, metabolic impairment, or membrane integrity as endpoints of the cytotoxicity test or assay to assess the short-term cytotoxicity of medical devices [138]. The objectives of the extraction test are to evaluate changes in cell morphology and growth inhibition, and determine whether cells are metabolically active [139].

An ISO guideline (10993- 5:2009) refers to the MTT cytotoxicity assay. MTT is a colorimetric method that measures the reduction of water-soluble yellow 3-(4,5-dimethylthiazol-2-yl)-2,5-diphenyl tetrazolium bromide by mitochondrial succinate dehydrogenase into an insoluble, blue-violet formazan. The number of viable cells correlates to the color intensity determined by photometric measurements after dissolving the formazan. The tested material is considered non-cytotoxic if the percentage of the viable cell is greater than or equal to 70% of the untreated control [124].

### 5.2. Direct Contact Assay

There are many limitations in the current tests used to check the effects of decellularized scaffolds on cells where the results can interfere with the presence of the biomaterial. It is important to understand that unleachable toxic substances that do not pass into the extraction medium can only be proven by direct cell contact [100]. In a direct contact assay, the sample is placed in direct contact with cells by surface culturing. Cells are examined at different time points for signs of toxicity by morphological examination and viability tests [140].

Most of the researchers preferred to test the cytocompatibility of decellularized scaffolds using direct contact assay, as it allows physiological changes made through the interactions of cells with a biomaterial, compared to the MTT assay, which only focuses on toxicity at a cellular level with less consideration of the molecular level [132].

## 6. Summary

This review summarized the application and advantages of the additive manufacturing (AM) technique via photo-polymerization 3D printing as a versatile platform in scaffold fabrication. Even though there are many techniques offered for scaffold fabrication, there are lots of characteristics and requirements that need to be considered in providing a scaffold with good mechanical, internal structure architecture, and biocompatibility properties. Photo-polymerization 3D printing has been reviewed as the most versatile technique and has the capability to produce a high accuracy dimensional architecture of a scaffold with flexibility in design.

Overall, an ideal selected fabrication TE scaffold should be carefully considered and capable of controling the variety in characteristic scaffold parameters needed in order to mimic the natural structure and properties of bone tissue.

## Figures and Tables

**Table 1:** Advantages and disadvantages of various fabrication techniques of 3D tissue engineering scaffolds.

Fabrication	Advantages	Disadvantages	Ref.
Techniques			
Solvent-casting and particulate- leaching	▪ Simple process ▪ Inexpensive ▪ Control porosity	▪ Limited size ▪ Low reproducibility ▪ Limited feature control ▪ Thickness < 4 mm ▪ Inefficient ▪ Poor mechanical properties	[17,18,38,39,40,41,42]
Gas foaming	▪ Control porosity ▪ Organic process	▪ Poor mechanical properties ▪ Imperfect pore ▪ Distinct structure ▪ Non-porous external surface	[17,42]
Phase separation	▪ Can combine with other fabrication technique ▪ Control porosity ▪ High porosity	▪ Complicated process ▪ Difficult control porosity ▪ Non-uniform porosity	[18,43,44,45]
Freeze drying	▪ Easy process ▪ Homogenous ▪ porosity ▪ Durable ▪ Flexible	▪ Small pore size ▪ Longer processing time ▪ Lower porosity	[39,42,46,47]
Fibre bonding	▪ High surface to ▪ volume ratio ▪ High porosity ▪ Easy process	▪ Poor mechanical properties ▪ limited applications ▪ Difficult control porosity ▪ Lack of solvent ▪ Complicated to set process parameters	[18,46]
Electro-spinning	▪ Low cost ▪ Flexible process ▪ Simple process ▪ Easy to find solvent ▪ Smooth fiber produced	▪ Low productivity ▪ Clogging problem ▪ Fragile fibers produced ▪ High-density nanofiber	[48,49,50,51,52]
Additive manufacturing (AM): rapid prototyping	▪ High accuracy ▪ High resolution ▪ Versatile scaffolds ▪ Homogenous cell distribution ▪ Interconnected pores ▪ Mimicking ECM ▪ Fast and easy process ▪ Custom made ▪ High reproducible ▪ No contamination ▪ Produces high cells density ▪ Conducted at room temperature ▪ Multi-color printing scaffolds ▪ Automated process ▪ Print scaffold with cells	▪ Lack of strength ▪ Limited raw materials	[53,54,55,56,57,58,59,60,61]

**Table 2:** Typical advanced manufacturing process for 3D tissue engineering scaffolds.

Fabrication Technique	Advantages	Disadvantages	Ref.
Photo-polymerization technique: stereolithography (SLA)/digital light processing (DLP))	Rapid response rate High-form precision Allows fabrication of internal pore scaffold Produces strong construction of complex tissue geometries scaffolds Cells can be incorporated Offers better dimensional dimensions Flexibility in design Higher accuracy and resolution compared to SLS/FDM/3D printing Resolution up to 100 nm Wide variety of application Able to create complex forms with the internal architecture Easily removes un-polymerized resin Versatile design	Requires photo-reactive biodegradable polymer in the process Ultraviolet irradiation used Produces layered stratification that may disable cell contact between layers Limited number of biocompatible resins because few bio- compatible polymers are stable under exposure to laser light Not suitable for high production rates due to the slow printing process	[65,66,67,68,69,70,71]
Fused deposition modelling (FDM)	Does not need any solvents and preserves flexibility in material handling and processing Highly controllable porosity Good mechanical properties Offers sufficient dimensional accuracy	Thermoplastic material used must have good melting viscosity properties Biomaterial used must be available in filament form Limited shape complexity for biological scaffolding materials Inability to incorporate living cells due to the high processing temperature during extrusion Insufficient surface	[36,58,68,72,73,74]
Selective laser sintering (SLS)	Able to produce complex shapes High mechanical strength Powder bed provides support for complex structures Fine resolution	Laser intensity can induce polymer degradation Limitation on materials (must be shrinkage and heat resistant) Trapped non-sintered material Poor control over surface topography High porosity Expensive and time consuming High-temperature process required	[73,74,75,76]

**Table 4:** The influences of resin selection and parameter setting for photo-polymerization 3D printing.

Input		Responses			
		Mechanical Properties	Bio-Compatibility	Porosity	Thickness Diameter
Resin used		[82,85,101]	[64,79,102]	[85,103,104]	[67,81,105,106,107]
Resin viscosity		[67,108]	[108]	[65,108,109]	[81,109]
Parameter setting	Curing time	[98,99,110]			
	Power light source				[81,100]
	Resolution				[81,110]
	Layer of thickness	[91,102]			
	Scan speed (velocity)				[81,100]

**Table 5:** Differences between polymer and resin [85,101,108].

Item	Polymer	Resin
Definition	Repeating structure unit of monomers	Organic material form in plant
Properties	Large molecular weight	Small molecular weight
Nature	Can be solid or liquid	Solid or highly viscous liquid

**Table 6:** Mechanical properties of various biomaterial resins for photo-polymerization 3D TE scaffolds.

Resin Materials	Filler/ Additive	Ratio	Photo Initiator	Ratio	Diluent	Ratio	Young’s Modulus (GPa)	Tensile Strength (MPa)		Porosity	Ref.
Poly(D,L- lactide) (PDLLA)	Fumaric acid mono ethyl ester (FAME)			5 wt%		35 wt%	n/a	n/a		76%	[116]
						NVP					
				6 wt%		0 wt%	Dry: 0.01	Dry: 1.30			
						NVP					
						30 wt%	Dry:	Dry:			
						NVP	1.50 ± 0.1	42.0 ± 4			
			Lucirin- TPO		N-vinyl-2- pyrrolidone (NVP)		Wet:	Wet:			
							0.80 ± 0.1	20.0 ± 3			
						40 wt%	Dry:	Dry:			
						NVP	1.80 ± 0.1	34.0 ± 10			
							Wet:	Wet:			
							0.80 ± 0.1	19.0 ± 1			
						75 wt%	0.02–0.2	20–70		95%	[108]
Poly (propylene fumarate) (PPF)	hydroxyl apatite (HA)	7 wt%	Bisacylph osphine oxide (BAPO)	1 wt%	Diethyl fumarate (DEF)	30 wt%	n/a	n/a		330 µm to 360 µm	[134]
				1 wt%		30 wt %	n/a	n/a		65%	[136]
		0 wt%					0.026 ± 0.001	0.6 ± 0.2			
ethylene	nano	0.3 wt%	Phenyl- 2,4,6- trimethylb enzoylpho sphinate (LAP)				n/a	1.2 ± 0.3			[137]
(PEGDA)		0.5 wt%					n/a	0.5 ± 0.1			
Poly(ethyle ne glycol) diacrylate (PEGDA)	Methyl methacryl ate (MMA)	50 %mol						32.68	2.78		[136]
	Butyl methacryl ate (BMA)	15 %mol									
	Methyl methacryl ate (MMA)	70 %mol						260.41	10.81		
	Butyl methacryl ate (BMA)	22 %mol									

**Table 7:** Advantages and disadvantages of biocompatibility tests [136].

Test	Advantages	Disadvantages
In-vitro tests	Fast testing Expensive Can be standardized Large-scale screening Good experimental control Excellence for mechanisms of interactions	Relevance to in vivo is questionable
In-vivo tests	Allows complex systemic interactions Responds more comprehensively than in-vitro tests More relevant than in-vitrotests	Expensive Time consuming Legal/ethical concerns Difficult to control Difficult to interpret and quantify
Usage tests	Relevance to use of the material is assured	Expensive Time consuming Difficult to control Difficult to interpret and quantify

## Data Availability

No new data were created or analyzed in this study. Data sharing is not applicable to this article.
